# Genome-Wide Identification of ABSCISIC ACID-INSENSITIVE (*ABI*) Genes and Their Response to MeJA During Early Somatic Embryogenesis in Longan (*Dimocarpus longan* L.)

**DOI:** 10.3390/plants14223508

**Published:** 2025-11-17

**Authors:** Muhammad Awais, Xiaoqiong Xu, Chunyu Zhang, Yukun Chen, Shengcai Liu, Yuling Lin, Zhongxiong Lai

**Affiliations:** 1 Institute of Horticultural Biotechnology, Fujian Agriculture and Forestry University, Fuzhou 350002, China; awais9518@gmail.com (M.A.); xuxq0921@163.com (X.X.); zcynhba@163.com (C.Z.); cyk68@163.com (Y.C.); lshc9264@fafu.edu.cn (S.L.); buliang84@163.com (Y.L.); 2 Key Laboratory of Genetics, Breeding and Multiple Utilization of Crops, Ministry of Education, Fujian Agriculture and Forestry University, Fuzhou 350002, China

**Keywords:** *Dimocarpus longan* Lour, somatic embryogenesis, abscisic acid-insensitive (*ABI*) genes, ROS, MeJA

## Abstract

Methyl jasmonic acid (MeJA) is a vital phytohormone that plays a key role in plant growth and adaptation to various environmental stresses. In the present study, on the basis of the longan genome, we identified a total of seven versatile putative abscisic acid-insensitive genes, which are the key players in plant growth and stress response. On the basis of bioinformatics analysis, transcriptome data, exogenous treatment experiments, and RT-qPCR findings, a comprehensive evolutionary pattern of *ABI* genes in different plant species and the effect of different MeJA treatments during early somatic embryogenesis in *D. longan* was carried out. The phylogeny results revealed that the seven *DlABI* genes evolved independently in monocots and dicots, having high protein sequence similarity, especially with Arabidopsis *ABI* genes. The comparative findings of gene structure, motif prediction, and synteny analysis suggest that *DlABI* genes disperse mainly through duplication events rather than localized tandem repeats. Furthermore, the correlations among the expressions of *DlABI* genes propose that the organization of the *cis*-regulatory elements in the promoter regions may regulate the temporal and spatial transcription activation of these genes. The qRT-PCR results revealed that the 50 µM MeJA treatment significantly upregulated the expression of *DlABI3*, followed by *DlABI1*, *DlABI2*, *DlABI5*, *DlABI4*, and *DlABI8,* respectively. The ROS findings clearly revealed that MeJA distinctly elevated the SOD, POD, and H_2_O_2_ activities while reducing catalase and MDA contents. The subcellular localization of *DlABI3* further confirmed its presence in the nucleus, suggesting its predicated transcriptional regulatory role in MeJA-mediated early SE in longan. Our findings reveal that the *ABI* genes are integral to the mechanism of MeJA-induced early somatic embryogenesis in longan by maintaining the ROS activity.

## 1. Introduction

Jasmonic acid (JA) is an endogenous growth-regulating chemical initially recognized as a stress-related hormone in higher plants [1]. JA and its derivatives, also known as jasmonates, are fatty acid-derived cyclopentanones that regulate growth and development, respond to various environmental stresses, affect gene expression, and play essential roles in plant defense responses [2]. There are several derivatives of jasmonic acid, such as jasmonoyl-isoleucine (JA-Ile), 12-hydroxy-jasmonic acid (12-OH-JA), 12-oxo-phytodienoic acid (OPDA), and methyl jasmonate (MeJA) [3]. Methyl jasmonate is recognized as a regulator that modifies physiological processes by influencing gene expression, ultimately impacting plant growth and development in response to environmental stress. Jasmonate regulates root growth inhibition and root hair elongation [4,5]. In addition to regulating gene expression by influencing gene-related transcription factors, MeJA plays a crucial role in the stress response and somatic embryogenesis, jasmonate signaling stress response, and metabolism [6,7].

*Dimocarpus longan* Lour., which is frequently known as the longan or “dragon eye” in Asia, originated from Southeast Asia and South China. It is an essential subtropical and tropical evergreen fruit tree that belongs to the Sapindaceae family [1]. Sapindaceae consists of 141 genera with approximately 1900 species, the majority of which are trees and shrubs. Four of these genera are economically essential, including *Litchi chinensis* Sonn., *Dimocarpus longan* Lour., *Nephelium*, and *lappaceum* L. China’s longan ranks first in terms of production and acreage. The origin of traditional medicine and the source of the edible drupe fruit, longan, is widely cultivated in many places in southern China, including Sichuan, Yunnan, Hainan, Guangdong, Guangxi, and Fujian [8].

In angiosperms, zygotic or sexual reproduction is the primary method of plant propagation. Sexual reproduction results in the formation of a seed that contains all the necessary components for seedling development [9]. Plant regeneration, germplasm conservation, and genetic advancement all depend on this mechanism. The five development stages of the SE process are induction, expression, maturation, germination, and transformation [10]. The groundwork for SE is laid by the induction phase. The type of explant used has a significant impact on the rate of induction. Compared to mature tissues like stems and leaves, immature zygotic embryos have been found to be the most effective explants because of their high induction rates [11,12]. Furthermore, SE induction is significantly influenced by the developmental stage of zygotic embryos, resulting in both callus induction and somatic embryo formation [13,14].

A critical plant tissue culture system, called somatic embryogenesis (SE), allows somatic cells to develop into new individuals via a pathway akin to zygotic embryogenesis, without undergoing the gametic phase [15]. Somatic embryos develop in much the same manner as zygotic embryos do. When somatic embryos are described in terms of their developmental stages of differentiation, they progress through the globular, heart, torpedo, and cotyledonary stages, ultimately becoming plantlets [12]. In-depth research is essential for *D. longan* embryo development, which has a significant effect on fruit production and quality [16]. There are several drawbacks to studying embryogenesis in its natural state, including poor synchronization and uncontrollable growth. To overcome these significant issues, the SE system established by Lai and others [17,18] has the potential to control growth and improve synchronization ability [19].

ABA-insensitive genes were isolated from *Arabidopsis thaliana* (L.) Heynh. Arabidopsis has genes that control the ABA response by encoding key transcription factors that are insensitive to ABA [20]. ABI1 and ABI2, the first *Arabidopsis* ABA response loci identified by mutation, were later found to encode highly homologous units of the PP2C family of ser/thr protein phosphatases [21]. The transcription factor called abscisic acid-insensitive 3 (ABI3) is well known to play a significant role in mediating plant stress tolerance [22]. ABI3 is a transcription factor that belongs to the B3 domain-containing protein family [23] and has four conserved domains, including three basic domains and one acidic domain. There is an acidic domain, A1, and three basic domains, B1s, B2s, and B3s [24]. The ABA-regulated AP2 domain transcription factor abscisic acid-insensitive4 (ABI4), which is mutated, increases the number of lateral roots (LRs) [25]. Thus, ABI4 plays a crucial role in coordinating the effects on (increasing the number of elongated) LR development, which is defined by the equilibrium between competing plant hormones [26]. Moreover, overexpression of ABI4 causes a decrease in the number of LRs, resulting in the inhibition of the elongation of emerged LRs [26]. Finkelstein’s team was the first to describe the consequences of an *ABI5* gene mutation. The Arabidopsis insertional mutant ABI5 was described as being less susceptible to ABA during seed germination than the wild type. The two chromosomes were the site of the mutation [27,28]. The ABA-insensitive8 (ABI8) mutant was discovered to be associated with resistance during germination. This mutant exhibit significantly poor stomatal control, stunted growth, altered ABA-responsive gene expression, suspended blooming, and male sterility [29]. It was clear from the molecular identification that the ABI8 locus is allelic to two dwarf mutants, known as eld1 (elongation defective 1) and kob1, in which hormonal abnormalities have yet to be discovered. This locus encodes a protein whose biochemical function is unexplored, suggesting that it may be part of a novel signaling pathway [30].

In the current study, we conducted genome-wide association and multiple experiments to elucidate the functional roles of key ABI transcription factors, which were classified on the basis of RNA sequencing data from MeJA treatment. The expression patterns of *DlABI* genes were determined and verified via qRT-PCR during the early stage of SE. The subcellular localization of *DlABI3* was also investigated to determine its role in longans’ further early SE and explore the potential relationship between abiotic stress and plant hormonal cross-talk. Although the *ABI* genes have been reported individually in various crop species [21,24], the information is scattered, and no comprehensive report on the *ABI* gene family has been published to date. The findings of the current study provide a foundation for versatile ABI TFs, which are crucial for plant growth and development, as well as their stress response.

## 2. Results

### 2.1. Identification and Physicochemical Characteristics of DlABI Genes

In the present study, we identified seven *ABI* (abscisic acid-insensitive) putative genes that encode ABI functions from three longan genomes and renamed them according to the nomenclature method used for *Arabidopsis ABI* genes. The *DlABI* genes were characterized for their physicochemical properties in the Supplementary Table S1. The encoded proteins possess distinct domain architectures and subcellular localizations. The domain of both *DlABI1* and *DlABI2* is PP2Cc, and their subcellular location is the chloroplast, whereas *DlABI3* has the B3 protein domain, which is predicted to be located in the nucleus. The protein domains and subcellular localizations of *DlABI4*, *DlABI5*, and *DlABI8* are AP2/ERF, bZIP, and glycotransferase-92, respectively. *DlABI4* and *DlABI5* are located in the nucleus, and *DlABI8* members are located in the chloroplast. The DlABI proteins exhibited considerable variations in their size (355–726 amino acids) and isoelectric points (5.51–9.2), indicating a considerable diversity in their biochemical characteristics. These findings will provide a basis for subsequent functional and expression studies related to *ABI* genes.

### 2.2. Phylogenetic Analysis of DlABI Family Members

**Figure 1:**
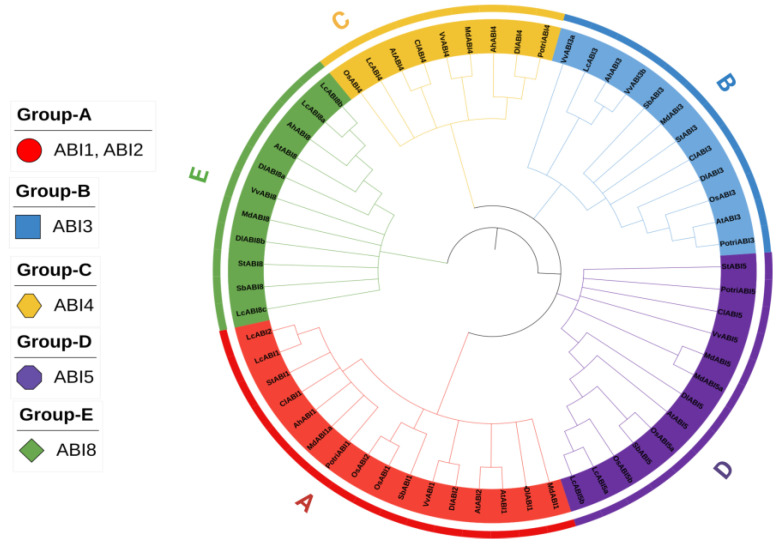
The phylogenetic tree of longan *ABI* genes, divided into five major clades named as *ABI1*, *ABI2*, *ABI3*, *ABI4*, *ABI5*, and *ABI8*.

To elucidate the evolutionary relationships and functional diversification of *DlABI* genes, a phylogenetic tree was reconstructed from a multiple sequence alignment of the conserved ABI protein domain. The current investigation incorporated 61 ABI protein sequences from a diverse set of plant species, including *Arabidopsis thaliana* (L.) Heynh., *Oryza sativa* subsp. *japonica* Kato, *Litchi chinensis* Sonn, *Malus domestica* Borkh., *Vitis vinifera* L., *Solanum tuberosum* L., *Populus trichocarpa* Torr. & A. Gray, *Arachis hypogaea* L., *Sorghum bicolor* (L.) Moench, and *Citrullus lanatus* (Thunb.) Matsum. & Nakai. The phylogenetic analysis characterized *ABI* genes into five well-supported clades (A–E), each of which corresponds to a specific ABI class defined by its domain architecture. The first specific class (Group A) contains a total of 16 *ABI-1* (abscisic acid-insensitive-1) and *ABI-2* (abscisic acid-insensitive-2) genes, which share a common protein domain named PP2C. Group B contains 12 *ABI3* genes, each having a B3 protein domain. The *ABI4* (Group C) and *ABI5* (Group D) included 9 and 13 genes, respectively, whereas the *ABI8* (Group E) comprised a total of 11 genes. The protein domain AP2/ERF belongs to the *ABI4* class, *bZIP* to the *ABI5* group, and *ABI8* is linked to the *glycotransferase-92* protein domain. It is clear from their topology that longan *ABI* genes share common ancestral origins with their orthologs in Arabidopsis, apple, and grape. Notably, a closer phylogenetic affinity was observed between longan and apple *ABI* genes that potentially reflects their shared status as woody perennial plants. Overall, the strong clustering of orthologous sequences within each clade demonstrates a greater degree of evolutionary conservation among ABI transcription factors across the monocot–dicot classes (Figure 1).

### 2.3. Chromosomal Location, Conserved Motifs, and Gene Structure of DlABI Family Members

**Figure 2:**
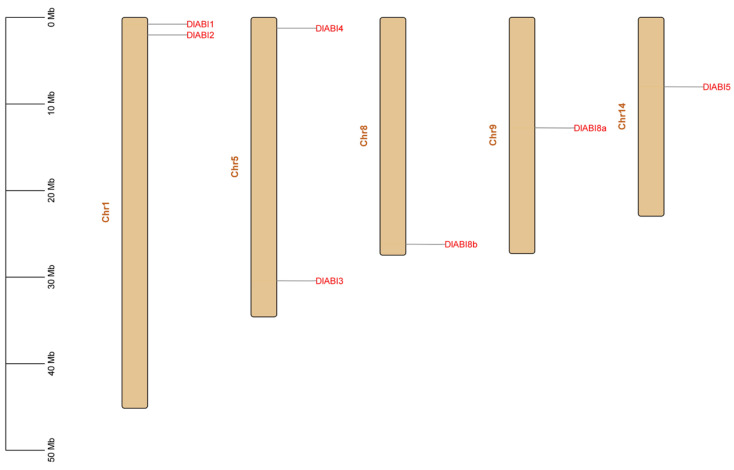
Distribution of *ABI* genes on Dimocarpus longan chromosomes. The scale bar on the left represents length (Mb).

The chromosomal localization results revealed that the seven identified longan *ABI* genes are unevenly dispersed across six different chromosomes, with no evidence of tight physical clustering (Figure 2). Specifically, *DlABI1* is located on chromosome 2, *DlABI2* is located on chromosome 1, and *DlABI3* and *DlABI4* are decentralized on chromosome 5, while *DlABI5*, *DlABI8a*, and *DlABI8b* are located on chromosome 9, chromosome 8, and chromosome 14, respectively. This distribution pattern suggests that the *DlABI* gene family members may have arisen from segmental or dispersed duplication events rather than tandem duplication.

**Figure 3:**
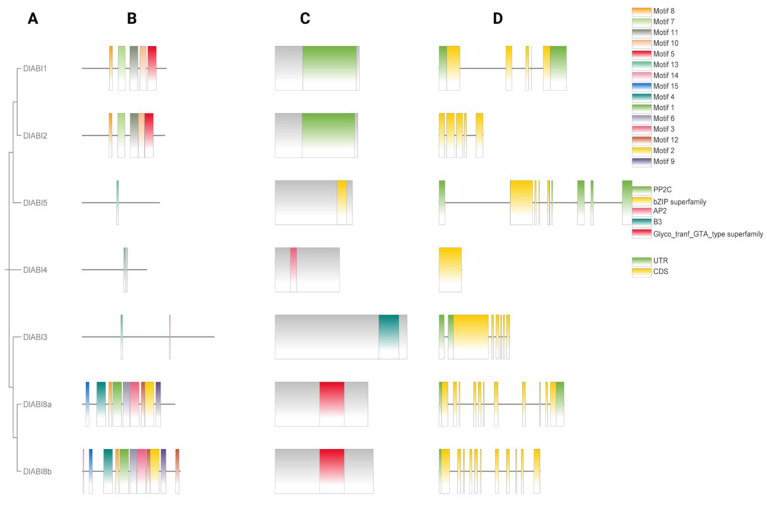
(**A**) Phylogenetic representation of longan *ABI* genes. (**B**) Motif illustration of longan *ABI* genes. (**C**) Depiction of protein domain analysis. (**D**) CDSs and UTRs of longan *ABI* genes.

The gene structure and conserved motif analysis were performed to further interpret the functional conservation and divergence of *DlABI* genes and to connect these molecular features with other layers of analysis, including phylogenetic classification, promoter composition, and expression profiles under MeJA treatment. Intron/exon formation was inferred through genomic DNA alignment and analysis of open reading frames (ORFs). The phylogenetic relationships of *DlABI* gene family members are presented in Figure 3A. The variety of protein domains is presented in Figure 3B. We searched 15 motifs in seven *DlABI* genes, which were consistent in type and order. *DlABI3* has motifs 13 and 14, whereas *DlABI5* only contains motifs 5 and 13. Specifically, motif nine is repeated in all genes, which may be a key regulatory element shared across all *DlABI* genes (Figure 3C). To further understand the basic characteristics, an exon–intron view was presented, comparing both the CDS and the UTR of seven putative ABI genes in longan. A comparison of both CDS and UTR intron size distribution regions revealed that the short introns were in the majority, indicating that the CDS intron length was more conserved than the UTR. Our findings disclosed that the UTR is absent in both *DlABI2* and *DlABI4* (Figure 3D). Overall, there were significant variations among *DlABI* family members, which may reflect evolutionary divergence among *DlABI* members.

### 2.4. Cis Element Analysis of DlABI Genes

**Figure 4:**
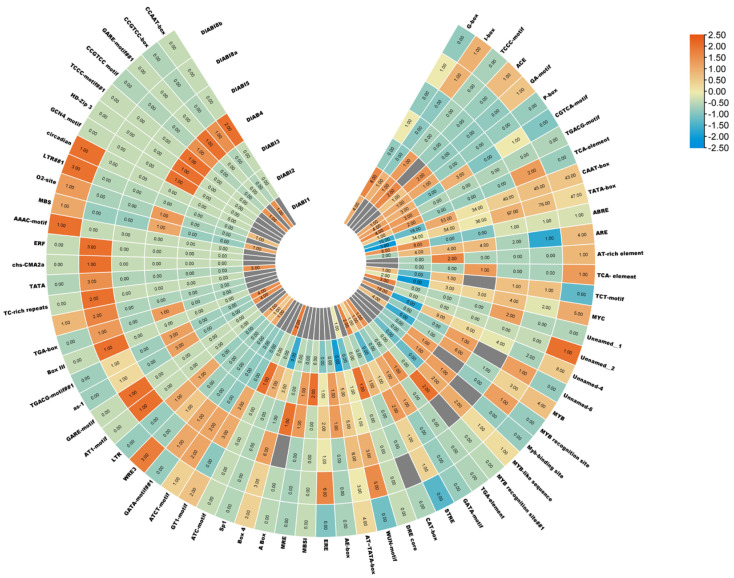
*Cis*-acting element analysis of *DlABI* genes. The 2 KB promoter sequences upstream of the *DlABI* initiation codon (ATG) of 7 *DlABI* genes were analyzed with PlantCARE v1 software.

To further evaluate the potential of *DlABI* genes and their response to hormones or environmental cues, the CREs (*cis*-regulatory elements) analysis results obtained through 2 KB promoter sequences upstream of the *DlABI* initiation codon (ATG) revealed a total of 1045 *cis* elements. We divided these elements into different categories and further subgroups in each class to express their specific roles. Our findings revealed that the numerous *cis*-acting elements in the promoter regions of *DlABI genes* were characterized as light-responsive, hormone-related, and promoter-associated. The class of light response-related *cis*-acting elements contains Box 4, G box, ATCT motif, GT1 motif, GATA motif, TCCC motif, ATC motif, TCT motif, GA motif, ACE, I box, Sp1, AE box, ARE, MYC, MYB, MYB-like sequence, Myb binding site, MYB recognition site, LTR, AT-rich element, MBS, STRE, MBS, MBSI, and DRE core. The plant growth and development class was further subdivided into meristem, metabolism, and circadian rhythm, while the hormone-related class contained abscisic acid responsive (ABRE), MeJA responsive (CGTCA motif, TGACG motif, TATC box), gibberellin responsive (p box, GARE motif, TATC box), auxin responsive (TGA-element, AuxRR-core), and ethylene responsive (ERE) *cis* elements in seven putative longan *ABI* genes. These cis-regulatory elements are the molecular bridge that connects gene expression to signaling molecules like ABA and MeJA. The *cis* elements affiliated with anaerobic induction, drought inducibility, low-temperature sensitivity, defense, stress responsiveness, and wound responsive element are enclosed in the stress-related category along with ARE, MBS, LTR, TC-rich repeats, WUN motif, and GC motif *cis* elements. The *cis* elements MBS1, O_2_-site, and MSA elements are linked to many aspects of metabolism, such as zein metabolism, palisade mesophyll cells, protein binding sites, cell cycle regulation, and flavonoid biosynthesis. The meristem expression’s CAT box and CCGTCC motif are examples of *cis* elements associated with meristems. Certain gene promoters contained endosperm-related *cis* elements, such as the GCN4 motif and AACA motif. These elements indicate potential regulatory roles of *DlABI* genes in hormones and stress-associated transcription, which further require expression verification (Figure 4).

### 2.5. Synteny Analysis and Chromosomal Duplication of DlABI Genes

**Figure 5:**
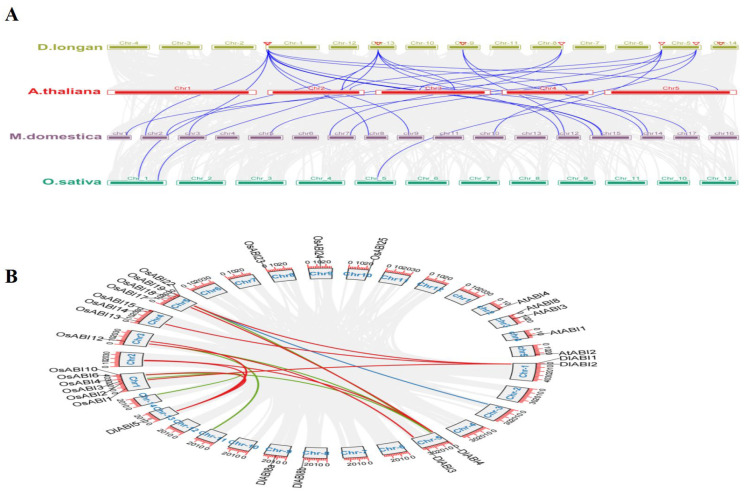
(**A**). Synteny analysis of *DlABI* genes between longan and the other three species. The colored lines highlight syntenic gene pairs of different gene families. (**B**). The circos plot between longan, Arabidopsis, and rice. The red, blue, and green colored lines depict collinearity.

To further investigate the gene-spot assemblage, latent genetic mechanisms, and functional characterization, we conducted a multiple genomic synteny analysis of *DlABI* genes in Arabidopsis, apple, and rice to elucidate their evolutionary history, origin, and potential functions. In terms of synteny analysis, gene duplication events that resulted in distinct chromosomes were classified as segmental duplications, whereas neighboring homologous longan *ABI* genes on a single chromosome, without a set of intervening genes, were classified as tandem duplications. Synteny analysis between longan and Arabidopsis revealed that the *DlABI* genes were widely distributed across three chromosomes, specifically on chromosomes 2, 4, and 5, except for *DlABI8a* and *DlABI8b*. Moreover, chr1 and chr13 of longan presented the syntenic attachments to chr1 of rice and longan chr5 with the chr5 of rice. Notably, the two gene members, *ABI1* and *ABI2*, were commonly on chromosome 1 in longan, Arabidopsis, and rice, indicating that these genes are multiple copies of the same gene in longan. *DlABI3* has multiple synthetic regions present on chromosome 3 of Arabidopsis and in apple, as well as on chromosomes 5 and 10. The *DlABI4* gene showed collinearity with Arabidopsis (chromosome 2), apple (chromosomes 1 and 5), and rice (chromosome 5). It is evident from the comparisons of longan and apple *ABI* genes that both tandem and segmental duplications occurred mainly on chromosomes 1 to 17. Two genes were clustered on chr2, chr7, chr12, chr14, and chr15, whereas single individual genes were clustered on chr1, chr3, chr8, chr10, and chr17 (Figure 5A). A circos plot of longan, arabidopsis, and rice was generated to further study the instinctive visualization of genomic distributions, evolutionary linkages, and structural features of *ABI* genes. The results revealed that several *DlABI* gene pairs derived primarily from segmental duplication events, while no evidence of tandem duplication was observed, which indicates that they likely resulted from a large-scale chromosomal duplication. Furthermore, integration of these findings with phylogenetic analysis, conserved motifs, and expression analysis provides a comprehensive understanding of the *DlABI* gene family’s evolutionary trajectory and functional relevance (Figure 5B).

### 2.6. Protein–Protein Interaction Network of DlABI Genes

**Figure 6:**
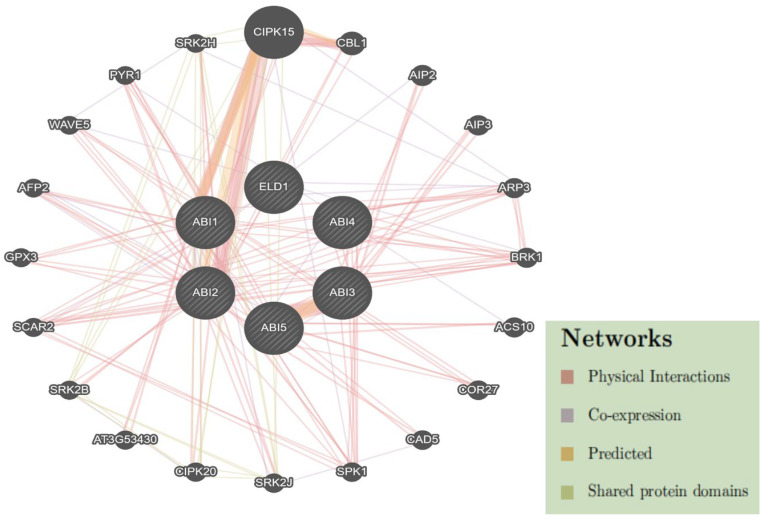
Protein–protein interactions network analysis of *DlABI* genes. Different parameters, including physical interactions, co-expression, predicted domains, and commonly shared protein domains, are labelled.

The bulk of proteins interact with other proteins to carry out the entire range of biological activities; however, many proteins also function independently, controlling every biological process within a cell. To explore the potential functional network and regulatory patterns of *DlABI* genes to the other interacting partners for mechanistic insight into how DlABI proteins integrate stress and hormonal signals, a protein–protein interaction network of *DlABI* genes based on *Arabidopsis thaliana* orthologous proteins via the online database GeneMANIA (https://genemania.org (accessed on 17 July 2025)) using default parameters. The detailed report has been provided in the Supplementary Data report. The analogous DlABI proteins, which are highly similar to those in *Arabidopsis thaliana*, were denoted as STRING proteins. All seven putative proteins related to *DlABI* were found to be associated with Arabidopsis proteins. Individual nodes represent all the proteins produced by a single cell or protein-coding gene locus, while the edges depict directly linked proteins that are part of the same physical complex (Figure 6).

### 2.7. Morphological Changes Assessment, Heat Map Expression Profiling, and the qRT-PCR Analysis of DlABI Genes in Response to MeJA Treatment

**Figure 7:**
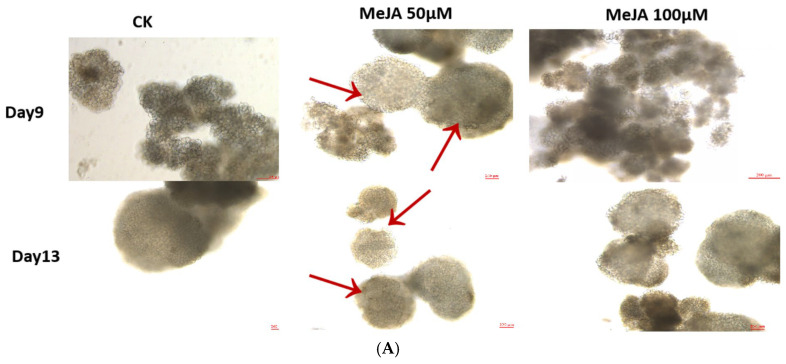

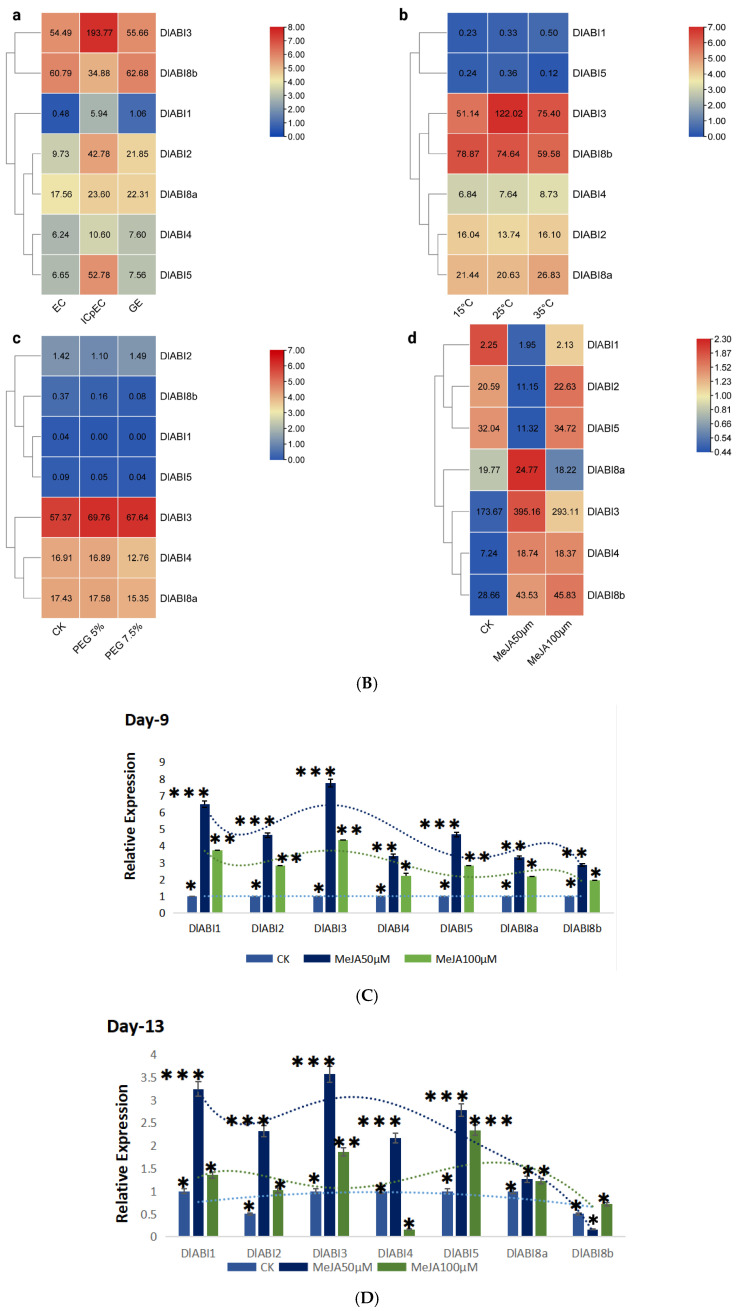
(**A**) Morphological assessment of longan ECs treated with 50 µM and 100 µM MeJA application with CK (control check) on the 9th and 13th days of treatment. The smoother and more compact cell edges are denoted with arrows. The scale bar of each image is 200µm (**B**) Heatmap generated from the RNA sequence data FPKM (log2 FPKM) of 7 represented *DlABI* genes. (**a**) Different callus stages. (**b**) Temperature conditions. (**c**) PEG treatments. (**d**) Different MeJA applications. (**C**) The relative expression of *DlABI* genes in response to MeJA 50 µM and MeJA 100 µM concentrations in comparison to control (CK) during early SE of longan determined by qRT-PCR on the 13th day. Values are the mean (*n* = 3) of three biological replicates, and the data is plotted as fold change (2^−ΔΔCT^) relative to control. The data were normalized to the UBQ gene. The *p*-value threshold of multiple comparisons is 0.05. Asterisks indicate significant differences (control vs. MeJA treatments’ comparison): “*” is *p <* 0.05, “**” is *p <* 0.01, and “***” is *p <* 0.001. (**D**) The relative expression of *DlABI* genes in response to MeJA 50 µM and MeJA 100 µM concentrations in comparison to control (CK) during early SE of longan determined by the qRT-PCR on the 13th day. Values are the mean (*n* = 3) of three biological replicates, and the data is plotted as fold change (2^−ΔΔCT^) relative to control. Asterisks indicate significant differences. The data were normalized to the UBQ gene. The *p*-value threshold of multiple comparisons is 0.05. Asterisks indicate significant differences (control vs. MeJA treatments comparison): “*” is *p* < 0.05, “**” is *p* < 0.01, and “***” is *p* < 0.001.

For a better understanding of *DlABI* genes, we analyzed the effects of MeJA treatment on morphological and expression pattern changes during early SE in longan. The results showed that with the progression in days, the MeJA-treated samples were in a more compact state in comparison to the control (CK), especially MeJA 50 µM. On the ninth day, the control samples were still in the pre-GE stage, but the MeJA-treated longan embryonic cells had formed early GE structures. Specifically, the 50 µM MeJA-treated cells were more wedged. The globular embryo formation with smoother cell edges was formed on the 11th day in 50 µM MeJA-treated calluses as compared to CK. The arrows point out the smoother and more compact cell edges, resulting in more stable early-GE formation in MeJA 50 µM. While the typical GE appeared after the 13th day in both control and treated samples, with a greater size of GE in the MeJA 50 µM treatment. Conclusively, it was found that the treatment with 50 µM MeJA significantly promoted the early somatic embryogenesis of longan, followed by 100 µM MeJA, in comparison to CK at 9 days after treatment. The morphological structure of embryogenic cultures showed distinct varying patterns between the CK and 50 µM MeJA groups. The cell morphology of both the CK and treated samples after the nine and thirteen days is presented in Figure 7A.

To further analyze the expression patterns of *ABI* genes in longan, the heatmaps were generated from the longan transcriptome data of our laboratory using fragments per kilobase million (log2 FPKM) values. All seven *DlABI* genes displayed for the three expression patterns EC (embryogenic callus), ICpEC (incomplete compact pro-embryogenic callus), and GE (globular embryos). The highest expression of *DlABI3* was observed at all three callus stages, whereas *DlABI1* had the lowest expression. Specifically, at the EC stage, *DlABI8b* showed higher expression, followed by *DlABI3*. Similarly, at the ICpEC stage, *DlABI3* had the greater expression patterns, followed by the *DlABI5* and *DlABI2* genes. At the globular embryo stage, *DlABI8b* and *DlABI3* presented the evidential expression. These findings suggest that the *DlABI* genes may play a critical part in promoting the process of longans’ early somatic embryogenesis (Figure 7A).

The RNA-sequencing data of *DlABI* genes treated with different temperature conditions, including 15 °C, 25 °C, and 35 °C, is presented in Figure 7B. It is evident from the findings of FPKM values that *DlABI8b,* followed by *DlABI3*, has the highest expression levels at 15 °C, whereas *DlABI1* and *DlABI5* showed the lowest expression. Similarly, at 25 °C temperature conditions, *DlABI3* recorded the highest expression, followed by *DlABI8b* and *DlABI1*, respectively. At more elevated temperature conditions (35 °C), the longan *ABI3* had the highest expression value, followed by *DlABI8b* and *DlABI5*, respectively. The varying expression patterns of *DlABI* genes from lower temperature conditions (15 °C) to elevated heat (up to 35 °C) suggest they may play a crucial role in regulating the process of embryogenic cell formation. To further elaborate on the effect of polyethylene glycol (PEG) on early somatic embryogenesis of longan, the heat map generated from the RNA sequencing data (FPKM) was presented with PEG 5% and PEG 7.5% in comparison to the control (Figure 7C). The findings revealed the highest expression level of *DlABI3* with both PEG 5% and PEG 7.5%, whereas the PP2C domain transcription factors *DlABI1* and *DlABI2* recorded the lowest expression levels when subjected to PEG treatments.

Further, we analyzed the expression patterns of seven *DlABI* genes under different concentrations of exogenous MeJA treatments at 9 and 13 days via qRT-*PCR* (Figure 7C). In general, expression was significantly up- or downregulated in both the MeJA 50 µM and MeJA 100 µM treatment groups compared with the control. On the ninth day, the expression of *DlABI3* in MeJA 50 µM was significantly upregulated, followed by *DlABI1*, *DlABI2*, *DlABI5*, *DlABI4*, and *DlABI8a*, respectively. *DlABI8b* showed downregulation on the ninth day in MeJA 50 µM. On the ninth day, MeJA 100 µM depicted significantly less upregulation in five *DlABI* genes in comparison with MeJA 100 µM treatment, except that *DlABI8a* and *DlABI8b* showed downregulation.

The expression patterns on the 13th day gradually downregulated in the MeJA 100 µM treatment and slightly upregulated in the MeJA 50 µM-treated longan EC. Specifically, the significant upregulation trend was clearly seen in *DlABI3*, *DlABI5*, and *DlABI8a* in 50 µM MeJA treatment, whereas a gradual downregulation trend was observed in the 100 µM MeJA group in all *DlABI* gene members except *DlABI3* and *DlABI1 (*Figure 7D). These findings proposed that the MeJA application could play a promising role in promoting SE by affecting the *DlABI* gene expression levels.

### 2.8. Subcellular Localization Investigation of DlABI3

**Figure 8:**
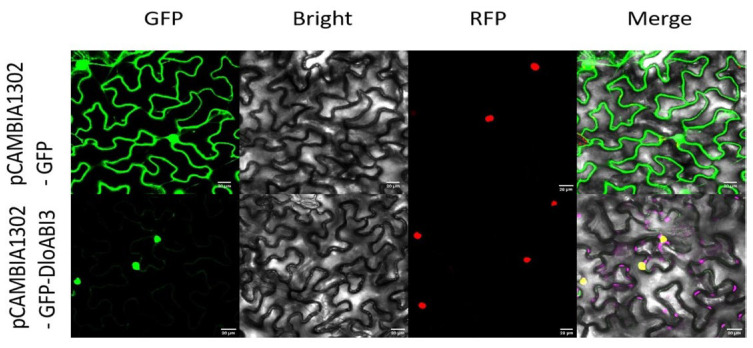
Subcellular localization analysis of *DlABI3*-GFP in tobacco leaves.

The sequence analysis, motif scanning, and domain annotation prediction outcomes suggested that ABI proteins act as nuclear transcription factors. Furthermore, *DlABI3* exhibited the highest expression patterns in the transcriptomic datasets presented in the current study; especially, a strong MeJA-induced expression during early SE was recorded. These findings support that the nucleus-localized *DlABI3* might be involved in mediating downstream responses. To further elucidate the potential role of *DlABI3* in longan, we selected *DlABI3* to determine its subcellular localization by using *DlABI3*-GFP fusion constructs. The predicted results from bioinformatics analysis indicated that *DlABI3* is localized in the nucleus. Tobacco leaves were used for confirmation of subcellular localization. The findings presented are consistent with the expected results, as the *DlABI3* location in tobacco leaves overlaps, with *DlABI3* located mainly in the nuclear region (Figure 8).

### 2.9. Determination of Reactive Oxygen Species (ROS) Enzymes Activity and Malondialdehyde (MDA)

**Figure 9:**
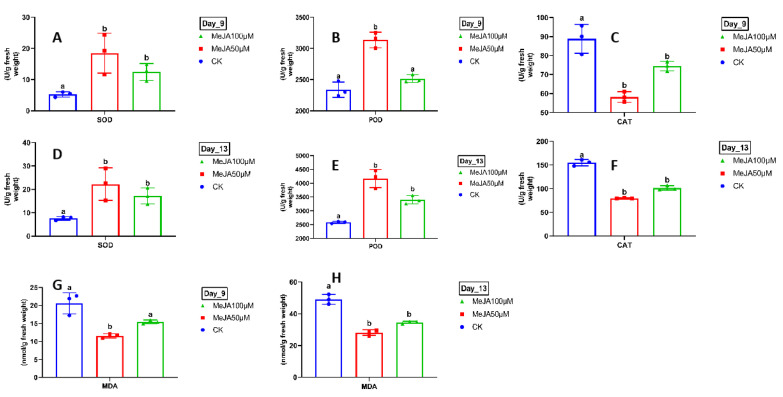
The measurement of reactive oxygen species (ROS) enzyme activity and malondialdehyde (MDA) in CK and MeJA treatment of longan Ec after the 9th day and 13th day. Data is the mean (*n* = 3) of three biological replicates with bars showing SD, and the lower-case letters above the bars indicate significant differences among treatments based on ANOVA followed by a *t*-test (*p* < 0.05). The ROS activities measured (**A**,**D**). SOD in (U/g fresh weight), H_2_O_2_ in (μmol/g fresh weight) (**B**,**E**). POD in (U/g fresh weight) (**C**,**F**). CAT in (U/g fresh weight) and (**G**,**H**) the malondialdehyde (MDA) in (nmol/g fresh weight).

To further clarify the oxidative status of longan embryogenic callus (EC) under MeJA treatments, the reactive oxygen species (ROS) and malondialdehyde (MDA) assays were performed. Our findings depicted a significant upsurge in the enzymatic activities, including superoxide dismutase (SOD), peroxidase (POD), and the hydrogen peroxide (H_2_O_2_) concentrations in longan EC treated with MeJA in comparison to the control, whereas catalase activity and MDA contents were recorded for the lower amounts in MeJA-treated EC in relation to CK (Figure 9A–H). MeJA is a pivotal signaling molecule that often stimulates redox imbalance as part of its regulatory roles in secondary metabolism and stress responses. Measuring the ROS provides deeper insights into the extent of oxidative burst triggered by MeJA, which may act as a secondary messenger to modulate *DlABI* gene expression and longans’ early SE, while malondialdehyde (MDA) is a stable end product of lipid peroxidation that primarily serves as an indicator of membrane damage caused by ROS.

## 3. Discussion

### 3.1. Evolutionary Conservation and Functional Diversity of the DlABI Genes

We identified the seven most pivotal *DlABI* genes, with special reference to the model plant *Arabidopsis thaliana*, which provides the foundation for five prominent transcription factor families: PP2C-type phosphatases, B3, AP2/ERF, basic leucine zipper (bZIP), and glycotransferase-92. Physicochemical investigations proved a broad spectrum of biochemical characteristics and cellular distributions for ABI proteins in longan. The multiple sequence alignment revealed high protein sequence similarity, suggesting that these genes may play overlapping functions in controlling gene expression. The phylogeny clustering demonstrates that the *ABI* genes underwent early evolutionary diversity well before the split between monocots and dicots. Phylogenetic clustering of *ABI* genes across different scaffolds indicates potential gene duplication events, a primary process by which gene families increase and diversify. It is evident from subcellular localization analysis that the three members, including *DlABI3*, *DlABI4*, and *DlABI5*, were located in the nucleus, while *DlABI1,2*, *DlABI8a*, and *DlABI8b* were found to be centralized in the chloroplast. Understanding the subcellular distribution of proteins can help elucidate their function. The presence of homology to a protein with a known localization is often a helpful indicator of the actual protein localization, as subcellular localization is evolutionarily conserved [31,32]. Phylogenetic relationships can be better understood through the study of gene architecture. Similar or identical numbers of exons and introns are found in the same subfamilies [33,34]. Through the three primary methods of exon/intron gain/loss, exonization/pseudoexonization, and insertion/deletion, the exon/intron diversification of gene family members significantly contributes to the development of numerous gene families [35,36]. The CDS and UTR elements present were also observed, as these elements are key regulators of gene expression. Chromosomes are dynamically rearranged during evolution through processes such as duplication, inversion, and translocation. The identification of homologous genes that maintain their ancestral locations (collinearity) facilitates comparative investigation of genomes. Synteny investigation facilitates the identification of collinearity blocks, and, in particular, by allowing multiple chromosome alignments, it supports evolutionary investigations [37]. *Cis* elements in the promoter region regulate gene expression, a mechanism that has emerged as the primary means by which organisms adapt to various environments [38,39]. Clues about the potential transcriptional regulation of genes can be found in the presence of *cis* element motifs in promoter regions [40].

### 3.2. DlABI May Regulate the Early Somatic Embryogenesis of Longan in Response to MeJA by Maintaining ROS Activity

In the present study, it is evident from genome-wide identification revealed by functional analysis that the *DlABI* genes might play a crucial role in the MeJA-induced early stage of SE in longan. RNA-seq analyses have been conducted to identify SE-related genes in various plant species, including *arabidopsis thaliana* (L.) Heynh. [40], *Oryza sativa* [41,42], and *Gossypium hirsutum* [42], to study the molecular regulatory mechanisms of plant SE. Prokaryotic and eukaryotic PP2C-type protein phosphatases are monomeric enzymes that control signaling pathways, development, and signal transduction [32]. The most conserved domain specific to the ABI3 is B3, and it has been found that many conserved cis elements in the promoter region of known seed-specific genes interact with domain B3 either alone, in conjunction with domain B2, or both [24]. Small RNAs, such as endogenous small interfering RNA (siRNA) and microRNAs (miRNAs), can regulate the expression of *ABI3* genes [42]. The transcriptional regulator ABA insensitive (*ABI4*) is essential for many physiological processes in plants, including sugar responses. However, little is known about the transcription factors that control sugar reactions and their function(s) in the signal transduction cascade [43,44]. *AP2/ERF* (APETALA2 ethylene-responsive factor) transcription factors regulate diverse developmental processes and stress responses in many plants, particularly in woody plants such as longan [45]. Furthermore, *ABI5*-related expression research supports its role in seedling development, enabling adaptation to salt and drought stress. *ABI5*-controlled suppression of seed germination and the early growth of seedlings guards against the development of plants under challenging circumstances [27]. However, the *ABI5* function is not limited to embryo tissues, and its role has also been described in the vegetative stage of development [44]. The *ABI8* locus encodes a protein whose biochemical function is unknown, suggesting that it may be part of a novel signaling pathway [46,47].

Phytohormones [48] specifically, jasmonates (JAs) and their methyl esters (MeJA) regulate plant growth, development, and responses to environmental stressors by interacting with enzymes, genes, and other growth regulators, thereby modulating signaling pathways and promoting the production of bioactive compounds [49]. A comprehensive report on the application and properties of jasmonates under in vitro conditions, including cell division, explant growth, proliferation ability, storage organ formation, and stress response, has been published [50]. The process of forming embryos from somatic cells is strongly influenced by stress conditions, including those induced by MeJA [51]. The two key phytohormones, ABA (abscisic acid) and JA (jasmonic acid), are key regulators of plant growth stress response, working synergistically or antagonistically depending upon their concentration [52].

*ABI* genes, especially *ABI5* (*ABI3* and *ABI4* in a broader context), are the nodes of JA -ABA cross-talk and are reported to be shown to respond to MeJA in non-SE, for instance, in *Arabidopsis*. The *ABI* interacts with JA pathway components; MeJA 50 µM alters ABI-related pathways and regulates the *ABI5* expression [53,54]. The specific dosage of MeJA (50 µM, 100 µM) used in the current study aligned with the previous reports, which clearly stated that these concentrations are established elicitors for embryogenic materials and modify gene expression, hormone levels, and metabolites. Like in Holm oak (*Quercus ilex* L.), where MeJA did not suppress the SE growth while changing hormone and phenolic profiles [54]. Similarly, the MeJA dose range of around 50 µM to 100 µM was reported to produce strong transcriptional responses and fluctuations in metabolites in rosemary (*Rosmarinus officinalis* L.) in vitro cultures [55]. The wayward roles of MeJA during SE have been widely reported in various studies. The involvement of MeJA in SE development of *Medicago sativa* has been reported. Unlike MeJA, the exogenous ABA probably inhibits the somatic embryo formation by altering ethylene biosynthesis [56]. Higher concentrations of MeJA in teak are reported to suppress the transition from globular and heart-shaped embryos to torpedo embryos. The critical insights into the complex interplay of various hormones, including MeJA, ABA, and SA, were expanded in the context of early somatic embryogenesis [57]. Our findings revealed that the low concentrations of MeJA treatment were found to be more effective in promoting early SE in longan. Similar reports have been found in the literature, where low dosage of methyl jasmonate acid are beneficial for somatic embryogenesis [58]. Under exogenous MeJA treatment, the development of somatic embryos was shown to be strengthened in saffron (*C. sativus*) [59]. Studies have shown that various environmental stresses can enhance the oxygen-induced damage due to the increased production of ROS, which causes lipid peroxidation, ultimately leading to membrane damage. A few reports have been published showing that hormones, including MeJA, induce oxidative stress in plants [60,61,62].

MeJA plays a critical role in eliciting the activity of ROS and maintaining the balance between hormonal cross-talk at various stages of plant development. Our findings revealed the critical upsurge in including SOD, POD, and the H_2_O_2_ activities and suppression in catalase and malonaldehyde contents. A previous study on the longan ABI5 transcription factor provides similar evidence, where MeJA was reported to be involved in the early SE and mitigating the temperature stress by stabilizing the ROS activities [63]. MeJA is not only involved in gene regulation but also plays a central role in enhancing secondary metabolites, especially lipid and lipid-like molecules, due to its chemical nature. A comprehensive report on the enrichment of secondary metabolites with various elicitors, including MeJA, has already been published [64]. A study conducted on plantlets of three different species, including *Galohimia glauca*, *Ruscus aculeatus*, and *Centella asiatica*, to boost the secondary metabolites with exogenous MeJA application revealed that a relatively high dose (100 μM) completely blocked the root growth in R. aculeatus with a notable upsurge in the secondary metabolites (triterpenoids) in *G. glauca*, followed by *C. asiatica* [65,66]. Various studies have shown that MeJA significantly affected secondary metabolite production, especially in Echinacea purpurea and Cymbopogon schoenanthus subsp. Proximus [67]. Similarly, the findings in citrus indicate a strong association between SE competence and the lipid accumulation of EC [68]. These findings suggest further exploring the potential roles of MeJA on metabolic profiles and pathways involved in early growth promotion of longan SE.

Nevertheless, there is no comprehensive report that has been published of *ABI* gene family members under MeJA treatments in fruit crops, especially in *longan*, despite the extensive work availability in model plants. Most of the previous studies have been carried out to elaborate the JA-responsive genes such as JAZ, LOX, and MYC, and the information on the crucial *ABI* genes is lacking. We presented the first combined report on genome-wide identification, transcriptomic data, RT-qPCR validation and ROS activities status in the context of *ABI* genes and their response to MeJA in longan, which will provide the basis to further elucidate the potential regulatory roles in linking MeJA-mediated *ABI* response in woody fruit crops, especially the Sapindaceae family.

## 4. Materials and Methods

### 4.1. Plant Materials

For the present study, we used the EC (embryogenic callus) of *D. longan* Lour. Honghezi (HHZ), which was established by Lai and others [18]. The longan SE involved three different developmental stages: embryogenic callus (EC), incomplete pro-embryogenic cultures (ICpECs), and globular embryo (GE) [19]. For the exogenous MeJA application, EC was transferred to MS (Murashige and Skoog) media provided by (Coolaber manufacturer Beijing, China) after 20 days of proliferation. The 50 µM MeJA and 100 µM MeJA concentrations were added to treat the materials for 13 days. The control check (CK) was used for comparison, and each treatment was replicated three times, containing 0.2 g of EC per replication. The somatic embryo differentiation status was observed using an optical microscope at 7, 9, 11, and 13 days. The samples were quickly transferred to liquid nitrogen and stored at −80 °C for subsequent analysis. Based on the comprehensive morphological assessment outcomes, a total of three groups were selected for RNA sequencing analysis. The control group samples were labeled as “control” (D), while the samples treated with the MeJA 50 µM group and the MeJA 100 µM samples were labeled as M210 and M215, respectively, each with three independent biological replicates.

### 4.2. Physicochemical Properties and Phylogenetic Analysis of DlABI Genes

Both the amino acid and genomic sequences of *Arabidopsis thaliana* (L.) Heynh., *Oryza sativa* subsp. *japonica* Kato, *Litchi chinensis* Sonn, *Malus domestica* Borkh., *Vitis vinifera* L., *Solanum tuberosum* L., *Populus trichocarpa* Torr. & A. Gray, *Arachis hypogaea* L., *Sorghum bicolor* (L.) Moench, and *Citrullus lanatus* (Thunb.) Matsum. & Nakai were downloaded from the public database Ensemble Plants FTP (ensembl.org) [69]. The whole genome sequences of longan were retrieved from the D. longan library (SRR17675476).

The accession numbers of the ABI amino acid sequences of four species, including *Arabidopsis thaliana* (L.) Heynh., *Oryza sativa* subsp. *japonica* Kato, *Vitis vinifera* L. and *Solanum tuberosum* L., were acquired from the literature and downloaded from NCBI. The Arabidopsis *ABI* genes used for the current study with their accession numbers are *AT4G26080.1*, *AT5G57050.1*, *AT3G24650.1*, *AT2G40220.1*, *AT2G36270.3* and *AT3G08550.1*.

The TBtools-II version 2.080 [70] was used by applying the bidirectional BLAST method for the identification of ABI genes. The arabidopsis protein sequences were used as bait to search the ABI of 10 species, including longan. The homologs of ABI genes were sorted out using parameters with an e-value < 1 × 10^−30^ and an identity higher than 40% and verified by using BLASTP. The resulting candidate proteins were then validated by scanning against the SMART, NCBI conserved domain database (CDD), and InterProScan database to confirm the presence and architecture similar to the AtABI proteins. The putative *DlABI* genes retained, which were confirmed from at least two independent domain prediction algorithms, were used for subsequent analysis.

The ExPASy tool (http://web.expasy.org/protparam/ (accessed on 17 July 2025)) was utilized to obtain the physicochemical properties [71]. The subcellular localization of *DlABI3* was predicted using WOLF PSORT (https://wolfpsort.hgc.jp/ (accessed on 17 July 2025)), and the protein was renamed according to Arabidopsis nomenclature [72]. The multiple sequence alignment of DlABI proteins was examined using MEGA 7.0 with MUSCLE progress. A maximum likelihood tree was constructed by using 61 amino acid sequences and bootstrap values of 1000 replications. The phylogenetic tree was annotated and visualized by utilizing iTOL version 6 “http://itol.embl.de/itol.cgi (accessed on 17 July 2025)” [73].

### 4.3. Domain Analysis and Gene Structure View of DlABI Genes

To predict the conserved motifs in longan *ABI* genes (with accession numbers *Dlo000068*, *Dlo000217*, *Dlo012591*, *Dlo010457*, *Dlo029652*, *Dlo020261*, and *Dlo019087*), the online software MEME (https://meme-suite.org/meme/tools/meme (accessed on 17 July 2025)) version 5.5.5 was used. It was used to set the range of motif numbers up to 10 and NCBI (https://www.ncbi.nlm.nih.gov/Structure/index.shtml, accessed on 15 July 2025). Tbtools software [70] was used for annotating chromosomal locations, gene structures, motifs, and conserved domains.

### 4.4. Analysis of Cis-Regulatory Elements and Synteny Visualization of Longan ABI Genes

To predict the cis-acting elements and gene co-expression concerning Arabidopsis thaliana in longan ABI genes, the Plant CARE database (https://bioinformatics.psb.ugent.be/webtools/plantcare/html/ (accessed on 17 July 2025)) was used. The genomic sequences were assessed in the promoter region and categorized based on their specific roles in predicting cis-acting elements of the *DlABI* genes promoter using Plant CARE [74]. For the synteny analysis between *D. longan*, *Arabidopsis thaliana* (L.) Heynh., *Oryza sativa* subsp. *japonica* Kato, and *Malus domestica* Borkh, the TBtools software (https://github.com/CJ-Chen/TBtools-II (accessed on 17 July 2025)) was used [70].

### 4.5. Analysis of the Specific Expression and the qRT-PCR Results of DlABI

The expression values of seven *DlABI* genes were extracted from RNA-seq data by using the FPKM for different treatments to longans’ early somatic embryos. For the early somatic embryogenesis stages (EC, ICpEC, and GE) and different light conditions (dark, white, and blue), the transcriptomic data of the following reference database [75] was used. Whereas for the PEG treatment and different temperature conditions [76], data was extracted and is provided in Supplementary Table S5. We used the DNAMAN6 software to design qRT-PCR primers for the longan *ABI* genes and TBtools for verifying primer specificity (Supplementary Table S1). Total RNA was extracted from MeJA-treated longan SE at 9 and 13 days after treatment by using the TransZol up reagent kit. For the conversion of total RNA into cDNA, the Revert aid Master Mix (Thermo Fisher Scientific, Shanghai, China) Kit was used for UBQ (UNIBIQUITIN) [77]. It was used as an internal reference. The Roche Light Cycler 96 was used to observe the expression levels. For qRT-PCR results analysis, GraphPad Prism 8 was used with one-way ANOVA. The three biological replicates were used, and the 20 μL reaction system contained the following: Super Mix (No Rox) (Heruibio, Guangzhou, China), 8.2 μL of ddH_2_O, 1 µL of 10-fold diluted cDNA, and 0.4 μL of specific primer pairs. The operating parameters of the qRT-PCR were as follows: 95 °C for 30 s, followed by 40 cycles of 95 °C for 10 s and 58 °C for 30 s. The relative expressions of *DlABI* genes were calculated using the 2^−ΔΔCT^ method [78,79].

### 4.6. The Subcellular Localization Analysis of DlABI3

For subcellular localization, the *DlABI3* was fused to the N-terminus of eGFP in the pBWA(V)HS-GFP vector, which was then transformed into the Agrobacterium strain GV3101 (provided by Bio Run Biotechnology Co., Ltd., Wuhan, China). Cells were grown overnight at 28 °C in a YEB medium supplemented with appropriate antibiotics. After centrifugation, the collected cells were resuspended in the MMA solution (10 mM MES, 10 mM MgCl_2_, 120μM acetosyringone) and incubated at room temperature for an additional 3 h on a shaker. The bacterium was suspended in the fresh buffer and adjusted to a final density of OD_600_ = 1.5. A needleless syringe was used to inject the suspensions into the abaxial side mesophyll cells of tobacco leaves. The treated plants were maintained in the greenhouse for 2–4 days before observation under a confocal microscope (Olympus FV1000, Olympus, Tokyo, Japan).

This study generated binary vectors for expressing GFP fusion proteins under the control of the 35S promoter. The vectors were cloned into pBWA(V)HS-GFP vectors and NLS marker proteins. NLS-mKate, a nucleus maker far-red fluorescent protein (mKate) with an N-terminal nucleus-localization sequence, was used. The vectors were transformed into Agrobacterium tumefaciens strain GV3101, mixed with the target gene and NLS marker plasmid, and assayed for fluorescence with a confocal laser scanning microscope (Olympus FluoView FV1000, Olympus, Tokyo, Japan) after 2–3 days of infiltration.

The binary vectors for the expression of the GFP fusion proteins under the control of the 35S promoter were constructed via LR reaction using the corresponding entry clones. The full-length *DlABI3* and NLS marker protein were cloned into the destination pBWA(V)HS-GFP vectors. All vectors were transformed in Agrobacterium tumefaciens strain GV3101 and, prior to infiltration, were resuspended in the MMA solution (10 mM MgCl_2_, 120 μM acetosyringone, and 10 mM MES) to OD_600_ = 0.8. Corresponding Agrobacterium strains containing the target gene with GFP constructs and the NLS marker plasmid (marker) were mixed 1:1 and co-infiltrated into the leaves of 2–4-week-old *N. benthamiana* plants. NLS-mKate, a nucleus marker far-red fluorescent protein (mKate) with an N-terminal nucleus-localized *DlABI3*, was used. The abaxial epidermis of infiltrated tobacco leaves was assayed for fluorescence by confocal laser-scanning microscopy 2–3 days post infiltration [80].

### 4.7. Measurement of Reactive Oxygen Species (ROS) Activity and Malondialdehyde (MDA)

The antioxidant enzyme activities were measured in both control and MeJA-treated longan EC. Firstly, longan EC (0.1 g) by fresh weight was ground in liquid nitrogen, and subsequently 1 mL of the extract was added, and the resulting homogenate was centrifuged at 800× *g* for a duration of 10 min at 4 °C. Finally, the supernatant was removed and placed on ice for testing. The collected supernatant was used for enzyme activity assays. For SOD, POD, CAT, H_2_O_2_, and MDA, the commercial kits (Keming, Suzhou, China) and a UV–visible spectrophotometer were used according to the manufacturer’s instructions [75].

## 5. Conclusions

**Figure 10:**
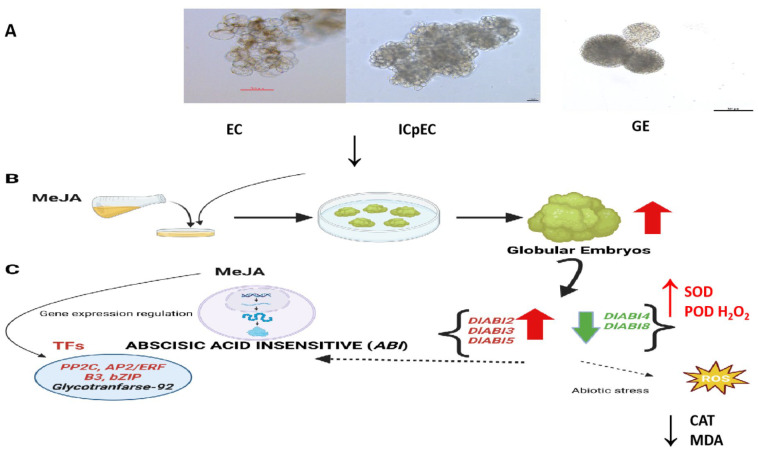
The molecular mechanism and regulatory network of *DlABI* during SE in longan. (**A**) The *DlABI* genes expressed during early somatic embryogenesis, i.e., embryogenic callus (EC), incomplete pro-embryogenic cultures (ICpCs), and globular embryo (GE). (**B**) *DlABI* in response to different MeJA concentrations greatly affected the GE (globular embryo) formation in longan. (**C**) The up- and downregulation expression of *DlABI* genes and related transcription factors that could participate in various hormonal pathways, ROS activity, and signal transduction ultimately affects the early stages of callus development in longan. The abbreviations used are MeJA (methyl jasmonic acid), SOD (superoxide dismutase), POD (peroxidase), CAT (catalase), H_2_O_2_ (hydrogen peroxide), and MDA (malondialdehyde).

The outcomes of the current investigation provide a basis for studying and classifying the versatile plant abscisic acid-insensitive (*ABI*) genes and perusing their response to MeJA during early somatic embryogenesis in longan. We have identified seven highly conserved *DlABI* genes via comprehensive bioinformatics analysis. The phylogenetic investigations depicted a clear association of individual *ABI* genes to five distinct gene families related to *ABI.* The chromosomal distribution, conserved motif, gene structure view, *cis*-acting elements, protein–protein interactions, and expression patterns of *DlABI* genes were analyzed. Overall, it was noticed that the lower concentrations of MeJA exert the promotive effect on longan EC, whereas high dosages restricted the cell proliferation of EC. The *DlABI* genes best responded to the MeJA 50 µM concentration. The specific ROS profiles suggested that MeJA imparts a promotive effect on longan’s early SE by modulating key components of the antioxidant system, fostering a feasible oxidative environment. Furthermore, the subcellular localization prediction of *DlABI3* was confirmed to be in the nucleus. Nevertheless, there were controversial views of MeJA in SE induction, and the regulatory mechanism is unclear in woody plants. This study will provide useful information to further explore the mechanism by which MeJA is involved in longan SE. Moreover, the current findings lay a foundation for future functional studies of MeJA-mediated ABA cross-talk as well as its application to other important plant species having challenging micropropagation systems. The expected model from the current findings is presented in Figure 10.

## Data Availability

All relevant data is available within the manuscript and Supplementary Materials.

## Supplementary Materials

The following supporting information can be downloaded at: https://www.mdpi.com/article/10.3390/plants14223508/s1. All the relevant data is contained within this article and the following: Supplementary Table S1: The physicochemical properties of *DlABI* genes. Table S2: Primers used for real-time quantitative PCR. Table S3: Primers used for subcellular localization of *DlABI3*. Table S4: *Cis*-acting element in the DlABI promoter region. Table S5: Log2 (FPKM) data table of *DlABI* genes in early somatic embryogenesis.

## Abbreviations

The following abbreviations are used in this manuscript:

Dl	Dimocarpus longan Lour
NEC	non-embryogenic callus
EC	embryogenic callus
ICpEC	incomplete compact pro-embryogenic culture
GE	globular embryos
SE	somatic embryogenesis
ABA	abscisic acid
ABI	abscisic acid-insensitive
TFs	transcription factors
CDS	coding sequence
UTR	untranslated region
ORF	open reading frame
PI	isoelectric point
MW	molecular weight
GA	gibberellin
SA	salicylic acid
qRT-PCR	real-time reverse transcription PCR
ROS	reactive oxygen species
SOD	superoxide dismutase
POD	peroxidase
CAT	catalase
MDA	malondialdehyde
