# Supplementary material for: Genome-Wide Identification of ABSCISIC ACID-INSENSITIVE (ABI) Genes and Their Response to MeJA During Early Somatic Embryogenesis in Longan (Dimocarpus longan L.)
Source: Plants (Basel). 2025 Nov 17;14(22):3508. doi: 10.3390/plants14223508 (PMC12656660; doi:10.3390/plants14223508)
Supplement: Supplementary file 1 [file plants-14-03508-s001.zip › Supplementary data report.pdf]

GeneMANIA report

Created on : 21 July 2025 22:51:50
Last database update : 13 August 2021 00:00:00
Application version : 3.6.0

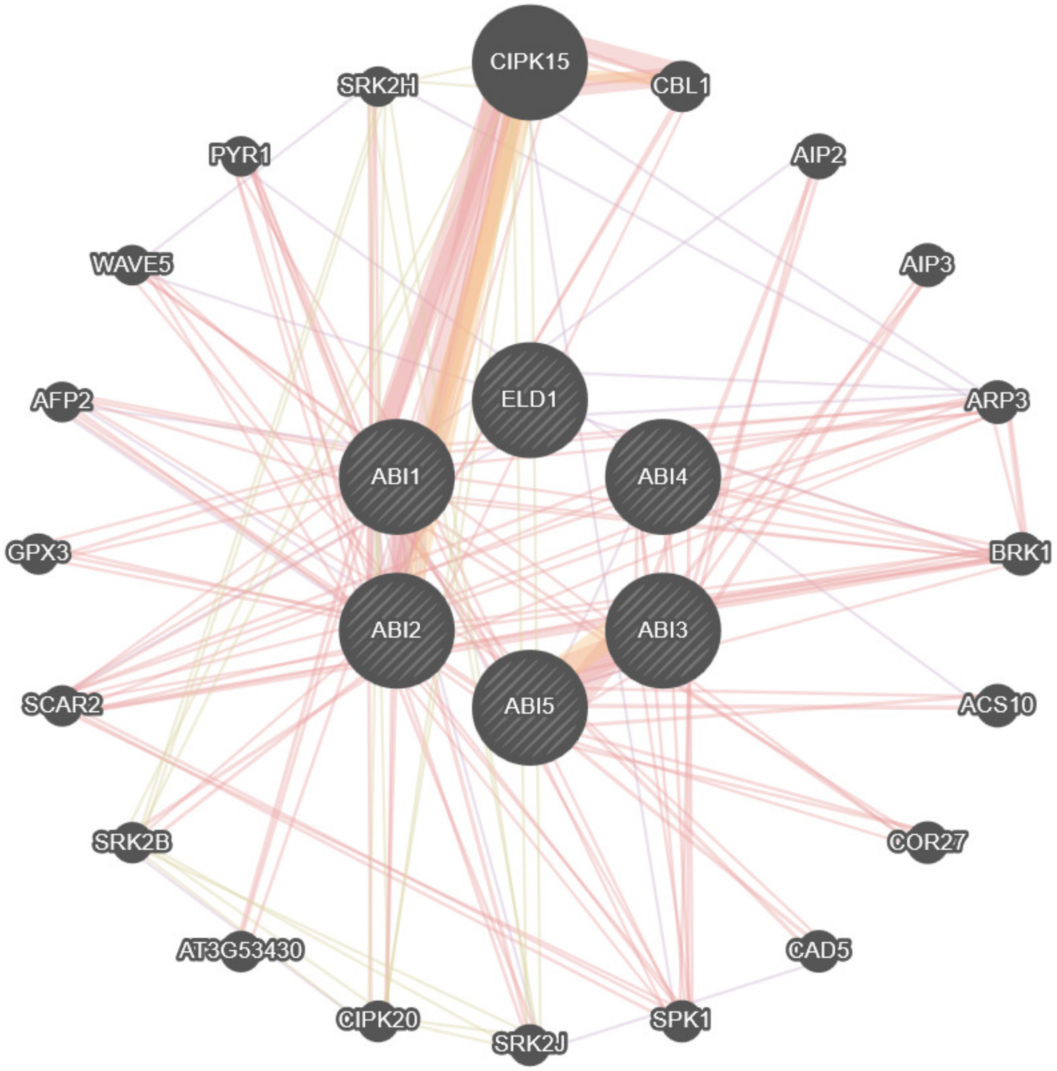

Networks

- Physical Interactions
- Co-expression
- Predicted
- Shared protein domains

Functions

N/A

Search parameters

Organism Arabidopsis thaliana (arabidopsis)

Genes ABI5 , ABI4 , ABI3 , ABI1 , ABI2 , ABI8

Network weighting Automatically selected weighting method

Networks

2

2011

A

Altmann-Falter-Braun-2020 , Arae-Chiba-2017

B

Bassel-Cutler-2008-Dormant Seed Expression , Bassel-Cutler-2008-Germinating Seed Expression , BIOGRID-SMALL-SCALE-STUDIES , BIOGRID-SMALL-SCALE-STUDIES , Boruc-Russinova-2010 A , Boruc-Russinova-2010 B , Brady-Benfey-2007 , Bustos-Paz-Ares-2010

C

Carianopol-Gazzarrini-2020 , Causier-Davies-2012 , Ceserani-Nelson-2009 , Chang-Bailey-Serres-2012 , Chen-Rhee-2012 , Cutcliffe-Rashotte-2011

D

Danisman-Immink-2013 , de Folter-Angenent-2005 , Deeken-Hedrich-2006 A , Dinneny-Benfey-2008 D , Dinneny-Benfey-2008 F , Dortay-Heyl-2008 , Dreze-Vidal-2011-combined , Dreze-Vidal-2011-main , Dreze-Vidal-2011-repeat

E

Efroni-Wagner-2013 , Elrouby-Coupland-2010

F

Fujiwara-Fukao-2014

G

Gifford-Birnbaum-2008 , Goda-Shimada-2008 A

H

Hackbusch-Uhrig-2005 , Hackenberg-Grimm-2012

I

Igawa-Yanagawa-2009 , INTERPRO , IREF-bar , IREF-bind , IREF-bind-translation , IREF-biogrid , IREF-dip , IREF-intact , IREF-intcomplex , IREF-mint , IREF-quickgo , IREF-SMALL-SCALE-STUDIES , IREF-uniprotpp

J

Jones-Frommer-2014

K

Kim-Vierstra-2013 , Klopffleisch-Jones-2011 , Kram-Carter-2009 , Kuroda-Matsui-2012

L

Lalonde-Frommer-2010 , Lee-Rhee-2010 AraNet , Lee-Rhee-2010 Co-citation worm2arabidopsis , Lee-Rhee-2010 Co-citation yeast2arabidopsis , Lee-Rhee-2010 co-expression human2arabidopsis , Lee-Rhee-2010 Co-expression worm2arabidopsis , Lee-Rhee-2010 Co-expression yeast2arabidopsis , Lee-Rhee-2010 Co-inheritance , Lee-Rhee-2010 Gene neighbourhoods , Lee-Rhee-2010 Genetic interactions worm2arabidopsis , Lee-Rhee-2010 Genetic interactions yeast2arabidopsis , Lee-Rhee-2010 Protein complexes human2arabidopsis , Lee-Rhee-2010 Protein complexes yeast2arabidopsis , Lee-Rhee-2010 Protein interactions , Lee-Rhee-2010 Protein interactions fly2arabidopsis , Lee-Rhee-2010 protein interactions from complexes yeast2arabidopsis , Lee-Rhee-2010 Protein interactions human2arabidopsis , Lee-Rhee-2010 Protein interactions worm2arabidopsis , Lee-Rhee-2010 protein interactions yeast2arabidopsis , Lee-Rhee-2010 Shared protein domains , Lee-Rhee-2010 shared protein domains human2arabidopsis , Lee-Rhee-2010 shared protein domains yeast2arabidopsis , Lee-Rhee-2010 Y2H human2arabidopsis , Lee-Rhee-2010 Y2H worm2arabidopsis , Lee-Rhee-2010 Y2H yeast2arabidopsis , Lumba-McCourt-2014

M

Manzano-Del Pozo-2008 , Mott-Belkhadir-2019 , Mukhtar-Dangl-2011 , Mustroph-Bailey-Serres-2009 B , Mustroph-Bailey-Serres-2009 C

P

Pandey-Albert-2010 , PFAM , Piya-Hewezi-2014 , Popescu-Dinesh-Kumar-2007 , Popescu-Dinesh-Kumar-2009 , PPI-Predicted (Interologs)

Q

Queval-Noctor-2012

R

Risseeuw-Crosby-2003 , Ronemus-Martienssen-2006

S

Sako-Yamaguchi-2014 , Schmid-Lohmann-2003 B , Schuler-Bauer-2011 , Smakowska-Luzan-Belkhadir-2018 , Swatek-Thelen-2011

T

Tamura-Hara-Nishimura-2010 , Tintor-Saijo-2013 , Trigg-Ecker-2017

V

Van Leene-De Jaeger-2010 , Van Leene-De Jaeger-2019 , Vernoux-Traas-2011

W

W

Waidmann-Jonak-2014

Y

Yamaoka-Hara-Nishimura-2013

Z

Zhang-Fernie-2018 , Zuber-Gallardo-2010 A

Genes

Gene	Description	Rank
ELD1	KOB1 [Source:UniProtKB/TrEMBL;Acc:A0A178V9W7]	N/A
ABI4	Ethylene-responsive transcription factor ABI4 [Source:UniProtKB/Swiss-Prot;Acc:A0MES8]	N/A
ABI3	B3 domain-containing transcription factor ABI3 [Source:UniProtKB/Swiss-Prot;Acc:Q01593]	N/A
ABI5	Basic-leucine zipper (bZIP) transcription factor family protein [Source:TAIR;Acc:AT2G36270]	N/A
ABI2	AtABI2 [Source:UniProtKB/TrEMBL;Acc:A0A178UGB7]	N/A
ABI1	Protein phosphatase 2C 56 [Source:UniProtKB/Swiss-Prot;Acc:P49597]	N/A
CIPK15	CBL-interacting serine/threonine-protein kinase 15 [Source:UniProtKB/Swiss-Prot;Acc:P92937]	1
CBL1	calcineurin B-like protein 1 [Source:TAIR;Acc:AT4G17615]	2
AIP2	E3 ubiquitin-protein ligase AIP2 [Source:UniProtKB/Swiss-Prot;Acc:Q8RXD3]	3
AIP3	Probable prefoldin subunit 4 [Source:UniProtKB/Swiss-Prot;Acc:Q9M4B5]	4
ARP3	Actin-related protein 3 [Source:UniProtKB/Swiss-Prot;Acc:Q9SAF1]	5
BRK1	Protein BRICK 1 [Source:UniProtKB/Swiss-Prot;Acc:Q94JY4]	6
ACS10	Probable aminotransferase ACS10 [Source:UniProtKB/Swiss-Prot;Acc:Q9LQ10]	7
COR27	Cold regulated protein 27 [Source:UniProtKB/TrEMBL;Acc:Q8L8T7]	8
CAD5	Cinnamyl alcohol dehydrogenase 5 [Source:UniProtKB/Swiss-Prot;Acc:O49482]	9
SPK1	Guanine nucleotide exchange factor SPIKE 1 [Source:UniProtKB/Swiss-Prot;Acc:Q8SAB7]	10
SRK2J	SNRK2.9 [Source:UniProtKB/TrEMBL;Acc:A0A178VSC8]	11
CIPK20	CBL-interacting serine/threonine-protein kinase 20 [Source:UniProtKB/Swiss-Prot;Acc:Q9FJ54]	12
AT3G53430	60S ribosomal Protein L12-like [Source:UniProtKB/TrEMBL;Acc:Q0WW72]	13
SRK2B	Serine/threonine-protein kinase SRK2B [Source:UniProtKB/Swiss-Prot;Acc:Q9C958]	14
SCAR2	Protein SCAR2 [Source:UniProtKB/Swiss-Prot;Acc:Q5XPJ9]	15

Gene	Description	Rank
GPX3	Probable glutathione peroxidase 3, mitochondrial [Source:UniProtKB/Swiss-Prot;Acc:O22850]	16
AFP2	AFP2 [Source:UniProtKB/TrEMBL;Acc:A0A178WBB1]	17
WAVE5	Scar-like domain-containing protein WAVE 5 [Source:UniProtKB/Swiss-Prot;Acc:Q5XPK0]	18
PYR1	Abscisic acid receptor PYR1 [Source:UniProtKB/Swiss-Prot;Acc:O49686]	19
SRK2H	Serine/threonine-protein kinase SRK2H [Source:UniProtKB/Swiss-Prot;Acc:Q9FFP9]	20

Networks

**Physical Interactions**	**50.95%**

IREF-bind — 19.74%

Physical Interactions with 670 interactions from iRefIndex

IREF-bind-translation — 19.74%

Physical Interactions with 678 interactions from iRefIndex

BIOGRID-SMALL-SCALE-STUDIES — 3.41%

Physical Interactions with 5,948 interactions from BioGRID

Lumba-McCourt-2014 — 2.47%

A mesoscale abscisic acid hormone interactome reveals a dynamic signaling landscape in Arabidopsis. Lumba et al (2014). *Dev Cell*

Physical Interactions with 379 interactions from BioGRID

IREF-bar — 2.28%

Physical Interactions with 8,121 interactions from iRefIndex

IREF-SMALL-SCALE-STUDIES — 1.94%

Physical Interactions with 7,477 interactions from iRefIndex

IREF-biogrid — 1.37%

Physical Interactions with 35,457 interactions from iRefIndex

**Co-expression**	**29.09%**

Deeken-Hedrich-2006 A — 4.26%

An integrated view of gene expression and solute profiles of Arabidopsis tumors: a genome-wide approach. Deeken et al (2006). *Plant Cell*

Co-expression with 609,443 interactions from GEO

Mustroph-Bailey-Serres-2009 C — 4.21%

Profiling translatomes of discrete cell populations resolves altered cellular priorities during hypoxia in Arabidopsis. Mustroph et al (2009). *Proc Natl Acad Sci U S A*

Co-expression with 803,119 interactions from GEO

Chang-Bailey-Serres-2012 — 4.19%

Transient MPK6 activation in response to oxygen deprivation and reoxygenation is mediated by mitochondria and aids seedling survival in Arabidopsis. Chang et al (2012). *Plant Mol Biol*

Co-expression with 683,527 interactions from GEO

Queval-Noctor-2012 — 2.63%

Day length is a key regulator of transcriptomic responses to both CO(2) and H(2)O(2) in Arabidopsis. Queval et al (2012). *Plant Cell Environ*

Co-expression with 657,861 interactions from GEO

Dinneny-Benfey-2008 F — 2.58%

Cell identity mediates the response of Arabidopsis roots to abiotic stress. Dinneny et al (2008). *Science*

Co-expression with 650,698 interactions from GEO

Co-expression 29.09%

Bustos-Paz-Ares-2010 2.41%

A central regulatory system largely controls transcriptional activation and repression responses to phosphate starvation in Arabidopsis. Bustos et al (2010). *PLoS Genet*

Co-expression with 691,702 interactions from GEO

Schmid-Lohmann-2003 B 2.11%

Dissection of floral induction pathways using global expression analysis. Schmid et al (2003). *Development*

Co-expression with 137,061 interactions from GEO

Goda-Shimada-2008 A 2.10%

The AtGenExpress hormone and chemical treatment data set: experimental design, data evaluation, model data analysis and data access. Goda et al (2008). *Plant J*

Co-expression with 684,584 interactions from GEO

Kram-Carter-2009 2.01%

Uncovering the Arabidopsis thaliana nectary transcriptome: investigation of differential gene expression in floral nectariferous tissues. Kram et al (2009). *BMC Plant Biol*

Co-expression with 672,952 interactions from GEO

Schuler-Bauer-2011 1.49%

Transcriptome analysis by GeneTrail revealed regulation of functional categories in response to alterations of iron homeostasis in Arabidopsis thaliana. Schuler et al (2011). *BMC Plant Biol*

Co-expression with 654,167 interactions from GEO

Pandey-Albert-2010 1.09%

Boolean modeling of transcriptome data reveals novel modes of heterotrimeric G-protein action. Pandey et al (2010). *Mol Syst Biol*

Co-expression with 689,348 interactions from GEO

Predicted 16.73%

PPI-Predicted (Interologs) 16.73%

A predicted interactome for Arabidopsis. Geisler-Lee et al (2007). *Plant Physiol*

Predicted with 18,163 interactions from collaborator

Shared protein domains 3.23%

INTERPRO 1.71%

Shared protein domains with 758,071 interactions from InterPro

PFAM 1.51%

Shared protein domains with 677,172 interactions from Pfam
